# From Inflammation to Fibrosis: Novel Insights into the Roles of High Mobility Group Protein Box 1 in Schistosome-Induced Liver Damage

**DOI:** 10.3390/pathogens11030289

**Published:** 2022-02-24

**Authors:** Haoran Zhong, Xiang Gui, Ling Hou, Rongxue Lv, Yamei Jin

**Affiliations:** 1 National Reference Laboratory for Animal Schistosomiasis, Shanghai Veterinary Research Institute, Chinese Academy of Agricultural Sciences, Shanghai 200241, China; 82101211282@caas.cn (H.Z.); 82101211645@caas.cn (X.G.); 82101211278@caas.cn (L.H.); 82101211248@caas.cn (R.L.); 2 Key Laboratory of Animal Parasitology of Ministry of Agriculture and Rural Affairs, Shanghai Veterinary Research Institute, Chinese Academy of Agricultural Sciences, Shanghai 200241, China; 3 College of Animal Science and Veterinary Medicine, Shanxi Agricultural University, Jinzhong 030031, China

**Keywords:** schistosomiasis, granulomas, high mobility group protein box 1, hepatic fibrosis, inflammation

## Abstract

Schistosomiasis is a chronic helminthic disease of both humans and animals and the second most prevalent parasitic disease after malaria. Through a complex migration process, schistosome eggs trapped in the liver can lead to the formation of granulomas and subsequent schistosome-induced liver damage, which results in high mortality and morbidity. Although praziquantel can eliminate mature worms and prevent egg deposition, effective drugs to reverse schistosome-induced liver damage are scarce. High mobility group box 1 (HMGB1) is a multifunctional cytokine contributing to liver injury, inflammation, and immune responses in schistosomiasis by binding to cell-surface Toll-like receptors and receptors for advanced glycation end products. HMGB1 is increased in the serum of patients with schistosomiasis and enables hepatic stellate cells to adopt a proliferative myofibroblast-like phenotype, which is crucial to schistosome-induced granuloma formation. Inhibition of HMGB1 was found to generate protective responses against fibrotic diseases in animal models. Clinically, HMGB1 presents a potential target for treatment of the chronic sequelae of schistosomiasis. Here, the pivotal role of HMGB1 in granuloma formation and schistosome-induced liver damage, as well the potential of HMGB1 as a therapeutic target, are discussed.

## 1. Introduction

Schistosomiasis, or bilharzia, is an infectious disease endemic in tropical areas of Asia, Africa, and South America affecting over 230 million individuals worldwide [1]. Three main species of schistosomes (i.e., *Schistosoma japonicum*, *S. haematobium*, and *S. mansoni*) account for most human infections [2]. *S. haematobium* is endemic in Africa, some areas of the Arabian Peninsula and was recently identified on the island of Corsica [3]. *S. japonicum* is prevalent in the Philippines, Indonesia, and China, while *S. mansoni* is endemic in Africa, the Middle East, and several countries in South America, including Brazil and Venezuela, and the Caribbean [4]. The young worms first pierce the skin and enter the blood vessels, then move through the heart and lungs to the liver vasculature, where mating and maturation of the male and female worms occur [5]. From there, the parasite couples migrate to the favored egg laying sites. *S. haematobium* migrates to the vesicle venous plexus, which induces urogenital schistosomiasis, while mating pairs of *S. japonicum* and *S. mansoni* migrate to the mesenteric vessels and induce intestinal/hepatic schistosomiasis [5,6].

Schistosome eggs are responsible for the occurrence and spread of schistosomiasis. Each mature female *S. mansoni* lays about 800 eggs per day, while female *S. japonicum* can produce up to 3000 eggs per day [7]. The enormous amounts of eggs deposited in the host’s liver by *S. japonicum* and *S. mansoni* trigger the primary pathology of schistosomiasis, which includes egg-induced granuloma formation, subsequent chronic inflammation, and eventual fibrosis due to the host immune response [8,9]. The extensive deposition of extracellular matrix (ECM) in the periportal areas is a typical symptom of hepatic fibrosis, giving rise to blockage of the portal veins, portal hypertension, splenomegaly, and gastrointestinal varices [2]. The pertinacious fibrosis of chronic schistosomiasis brings about hepatic cirrhosis and is responsible for the high mortality rate of liver cancer in Asian countries [10,11]. In the advanced phase, the course of schistosome-induced liver diseases is often irreversible [12]. Although the chemotherapy agent praziquantel (PZQ) can effectively eliminate mature worms and prevent the accumulation of eggs, there is a lack of effective drugs directly targeting and reversing schistosome-induced hepatic fibrosis [13]. Without prompt treatment, liver fibrosis can further transform into portal hypertension and ascites, which are usually the main causes of death in patients with chronic schistosomiasis [14]. Therefore, the development of new treatments targeting factors that regulate hepatic fibrosis in schistosomiasis patients remains a priority.

High mobility group box 1 (HMGB1) is a highly conserved DNA-shepherding protein that is plentiful in the cell nucleus [15]. HMGB1 is actively secreted by multiple cell types, including macrophages, monocytes, dendritic cells, natural killer cells, endothelial cells, and platelets [16], and passively by necrotic and damaged cells [17,18]. Either mode can release substantial amounts of extracellular HMGB1, which participates in multiple biological functions. Serum and liver levels of HMGB1 are significantly increased in some schistosomiasis patients with inflammatory responses, suggesting a close association with disease progression [19]. Moreover, recent studies suggest that HMGB1 is significantly upregulated in patients with schistosome-induced hepatic fibrosis as well as animal models [20]. Meanwhile, inhibition of the translation and release of HMGB1 or blocking related signaling pathways via the HMGB1 receptor can protect against hepatic inflammation and fibrosis, suggesting that HMGB1 may be a vital factor in schistosome-induced liver disease as well as a potential therapeutic target [20,21,22]. Accordingly, targeting of HMGB1 could potentially reduce the occurrence and pathogenesis of schistosome-induced liver disease, although the underlying mechanism remains unclear.

Therefore, based on the extensive body of literature, the present review briefly summarizes the structure and functions of HMGB1, the development and migration of schistosomes in the host, factors that contribute to the regulation of schistosome-induced liver disease, the crucial role of HMGB1 in schistosome-induced liver disease, and HMGB1 as a potential target for treatment of schistosomiasis-associated inflammation and fibrosis.

## 2. HMGB1 Structure and Receptors

### 2.1. HMGB1 Structure

**Figure 1:**
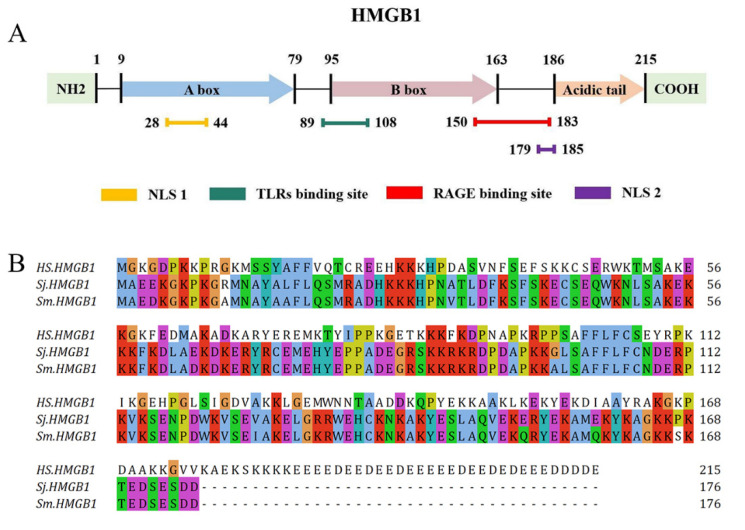
Structure of human HMGB1 and the complete HMGB1 protein sequence of *Homo sapiens*, *S. japonicum* and *S. mansoni*. (**A**) Human HMGB1 consists of 215 aa residues, which can be divided into the A box (aa 9–79), B box (aa 95–163), an acidic tail (aa 186–215), two NLSs (aa 28–44 and aa 179–185), a TLR binding site (aa 89–108), and a RAGE binding site (aa 150–183). (**B**) The complete protein sequence of *H. sapiens* HMGB1 (GenBank accession no. NP_002119) in relation to the orthologues of *S. japonicum* (DQ005528) and *S. mansoni* (DQ005529).

HMGB1, first isolated and identified in 1973 by Goodwin et al. [23], is a non-histone chromosome-binding protein composed of 215 amino acid (aa) residues and is named to describe the high electrophoretic mobility in polyacrylamide gels. The structure of human HMGB1 is composed of two functional DNA-binding domains, the A box (aa 9–79) and B box (aa 95–163), an N-terminus, and a C-terminus, known as the “acidic tail”, which consists of 30 glutamic and aspartic acid residues [24] (Figure 1). The sequence of HMGB1 is 98.5% identical in all mammals. The B box is the functional region of HMGB1 that promotes inflammation, while the A box is the antagonistic site that can competitively inhibit the pro-inflammatory effect of the B box [18]. Besides, HMGB1 carries two nuclear localization signals (NLSs): NLS 1, which is located in the A box (aa 28–44), and NLS 2, located in the B box (aa 179–185) [25]. Moreover, HMGB1 contains a Toll-like receptor (TLR) binding site (aa 89–108) and a receptor for advanced glycation end-products (RAGE) binding site (aa 150–183) [24]. Interestingly, the HMGB1 sequences of *S. japonicum* (SjHMGB1) and *S. mansoni* (SmHMGB1) are identical and share 60% similarity with mammalian HMGB1. Unlike human HMGB1, which contains an unbroken run of 30 glutamic or aspartic acid residues, SmHMGB1 and SjHMGB1 possess unusually short acidic C-terminal tails, composed of five acidic residues interrupted by two serine residues [26].

### 2.2. HMGB1 Receptors

RAGE, a transmembrane protein of the immunoglobulin superfamily, is expressed on the surfaces of monocytes, macrophages, endothelial cells, vascular smooth muscle cells, and neurons [27]. RAGE expression is higher in most cell types and tissues in a disease state as compared with a healthy state [28,29,30]. In addition to HMGB1, RAGE interacts with many other ligands, such as advanced glycation end products, members of the S100 protein family, and beta-amyloid peptide [28], which further triggers activation of the Ras-extracellular signal-regulated kinase 1/2 (ERK1/2) pathway [31], stress-activated protein kinase/c-Jun-NH2-terminal kinase (SAPK/JNK) pathway, Cdc42/Rac pathway [32], and p38 mitogen-activated protein kinase (MAPK) pathway [30]. Finally, the proinflammatory effects of HMBG1 can be amplified through signaling cascades of transcription factors, such as nuclear factor (NF)-κB [33], cAMP response element-binding protein [31], and members of the signal transducers and activators of transcription family with the involvement of the RAGE ligand [34]. Accumulating evidence suggests that activation of RAGE signaling in the liver can lead to the development of numerous types of hepatic disorders [35]. For example, Gaens et al. [36] found that fatty acids could stimulate Nε-(carboxymethyl) lysine accumulation in hepatocytes and subsequently elicit inflammatory reactions and hepatic steatosis via the induction of RAGE, while Nomota et al. [37] suggested that the lack of galectin-3 was related to the progression of non-alcoholic fatty liver disease in mice, which was also associated with up-regulated RAGE expression in hepatocytes. 

TLRs are type I transmembrane pattern recognition receptors that can identify diverse signals, such as damage-associated molecular patterns and pathogen-associated molecular patterns [38]. TLRs have been identified as intermediary players linking the innate and adaptive immune responses in favor of initial enhancement of inflammation [39,40,41]. In gastric ischemia-reperfusion injury, HMGB1 interacts with TLR2, which leads to significant organ damage after reperfusion [42]. Meanwhile, Azam et al. [43] found that myeloid differentiation primary response 88 (MyD88)- or non-MyD88-dependent signaling could be activated by HMGB1 to trigger downstream signals via activation of TLR4, which enhances the secretion of cytokines via the NF-κB pathway. In addition, HMGB1 has been associated with activation of the TLR9/MyD88 pathway and the release of proinflammatory cytokines in autoimmune thyroiditis [44]. Besides, the lack of HMGB1 leads to increased susceptibility to hepatocyte death through TLR9 in response to oxidative stress [22]. In summary, TLRs participate in the signal transduction of HMGB1, which further promotes the transcription of cytokines and chemokines via activation of inflammatory signaling pathways.

## 3. Schistosome-Induced Liver Damage

### 3.1. Life Cycles of Schistosomes

The entire life cycles of schistosomes are key to the occurrence and spread of schistosomiasis. The life cycles of *S. japonicum*, *S. mansoni*, and *S. haematobium*, the three main species of schistosomes that infect humans, slightly differ according to distinct egg-laying sites, as well as migration inside the host [45,46]. 

The release of *S. japonicum*, *S. mansoni*, and *S. haematobium* cercariae from the intermediate host snails (freshwater Oncomelania, Biomphalaria, and Bulinus spp., respectively) breach the epidermis as the first step of infection [6]. Almost 90% of *S. japonicum* can penetrate the dermal vessels within 2 h after infection and reach the dermis within 24 h. However, the migratory pattern of *S. mansoni* and *S. haematobium* significantly differ from that of *S. japonicum*. At 72 h after infection, the majority of *S. mansoni* and *S. haematobium* schistosomula reach the dermal vessels [47,48]. 

As the second step of the migration process, the schistosomula travel through the circulation to the lungs. Due to the small diameters of pulmonary capillaries, the young parasites elongate to facilitate penetration [49]. *S. japonicum* and *S. mansoni* schistosomula are detected in the lungs between 2–3 days post infection (dpi), with *S. japonicum* peaking at 3 dpi and *S. mansoni* at 7 dpi [50,51]. On the other hand, *S. haematobium* can reach the lungs at around 7 dpi and remain for up to 25 dpi [52].

As the third step of the migration process, schistosomula can travel from the lungs to the systemic organs through the pulmonary veins. The descending (abdominal) aorta, gastric arteries, gastroduodenal artery, or splenic artery may be the direct or indirect route for schistosomula to reach the hepatic portal vein [5]. To facilitate maturation, schistosomula use ventral suckers to crawl on the branches of the hepatic portal vein to feed on blood [53]. Upon maturation of the young parasites to adults, which occurs in about 5–7 weeks, the worms form mating pairs and migrate from the liver of the host to the preferred sites to lay eggs [6].

As the fourth stage of the migration process, adult mating pairs of *S. japonicum* and *S. mansoni* migrate to the venous vasculature close to the intestines and lay eggs [50,51]. The eggs penetrate the blood vessel wall to reach the intestinal lumen and are excreted in feces to complete the migration process in the host [5]. Previous autopsy studies of humans infected with *S. japonicum* or *S. mansoni* reported that most parasites were widely distributed in the liver, hepatic portal vein, and mesenteries, which were considered their natural habitat [54,55]. Due to the inexorable exploratory nature of intravascular schistosomes, eggs have also been discovered in nonproductive sites, which include the spleen [54], stomach [56], pancreas [57], gall bladder [57], ovary [58], fallopian tube [59], prostate [60], brain [61], and spinal cord [62]. Interestingly, SjHMGB1 is abundantly expressed in the adult and egg phases, but barely detectable in the cercaria stage [63]. However, further studies are needed to elucidate the involvement of HMGB1 in the development of *S. japonicum*.

Unlike *S. japonicum* and *S. mansoni*, *S. haematobium* primarily migrates from the hepatic portal vein to the vesicle venous plexus surrounding the bladder and induces urinary schistosomiasis [5]. In women, *S. haematobium* eggs have been found in the cervix, uterus, vagina, fallopian tubes, and ovaries, which all contribute to female genital schistosomiasis [64,65]. In men, eggs released by *S. haematobium* have been found in the epididymis, prostate, seminal vesicles, vas deferens, and testes, leading to male genital schistosomiasis [66]. Eggs in the vesicle venous plexus can penetrate the blood vessel and the wall of the bladder, then enter the bladder lumen and finally exit the body in urine [5,6].

Once eggs are excreted from the host (*S. japonicum* or *S. mansoni* in feces and *S. haematobium* in urine), miracidia hatch from the eggs in freshwater and look for a suitable snail as an intermediate host to complete and repeat the life cycle.

**Figure 2:**
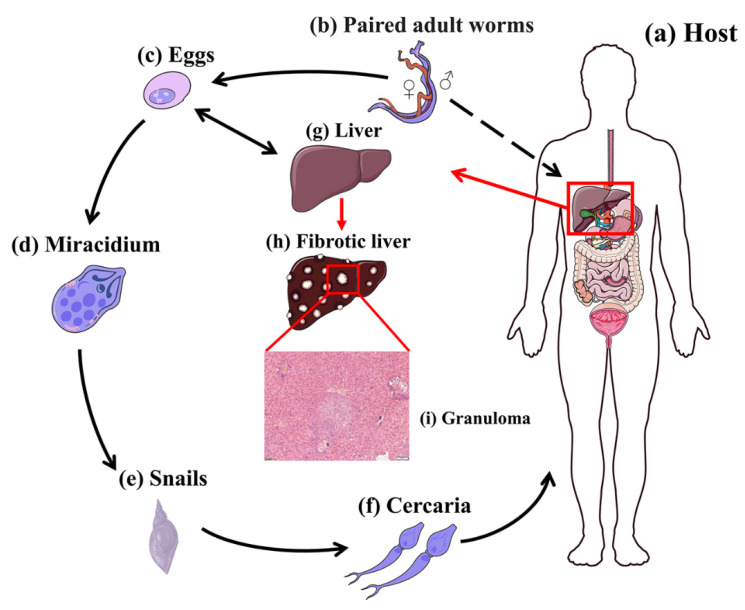
Lifecycle of *S. japonicum*. (**a**) Host. (**b**) Paired adult worms (larger male enfolding slender female). (**c**) Eggs. (**d**) Miracidium. (**e**) Intermediate host Oncomelania. (**f**) Cercariae. (**g**) Healthy liver. (**h**) Fibrotic liver with granuloma. (**i**) Representative histology of *S. japonicum*-induced granuloma formation in BALB/c mice at 6 weeks post-infection. Formalin-fixed, paraffin-embedded liver sections stained with hematoxylin and eosin (H&E). Scale bars, 100 mm.

Nevertheless, about one-third to one-half of eggs released by schistosomes fail to reach the external environment but rather become trapped in various tissues of the host, such as the liver, where the eggs induce a potent inflammatory response, leading to the formation of granulomas, the most basic and crucial pathologic form of schistosomiasis [67]. The life cycle of *S. japonicum* and the process of egg-induced granuloma formation are illustrated in Figure 2.

### 3.2. Formation of Liver Granulomas

Schistosome eggs have highly antigenic structures that constantly secrete various types of toxic or antigenic substances into the tissues of host [68]. Evidence suggests that schistosome eggs, but not adult worms, induce the pathogenicity of schistosome infections [2,6]. Granulomas and subsequent hepatic fibrosis occurring in the middle and late stages of infection result from the host immune response to the soluble egg antigen of schistosome eggs [69,70]. 

The dynamic development of liver granulomas can be segmented into acute and chronic phases based on the interactions of various cell types, including macrophages, neutrophils, eosinophils, and hepatic stellate cells (HSCs) [9,71]. Generally, the early acute phase of schistosome infection is regulated through T-helper (Th)1 responses via the up-regulated expression of the Th1 proinflammatory cytokines interleukin (IL)-1, IL-12, interferon (IFN)-γ, and tumor necrosis factor (TNF)-α, which targets the migrating schistosomula and mature adult worms [72]. In the chronic phase (6–8 weeks post infection), the immune response gradually switches to a Th2 response as the schistosome eggs are deposited [5,72]. The switch from the Th1- to Th2-mediated immune response can protect the host against the lethal outcomes of chronic, unregulated Th1 proinflammatory responses [73].

**Figure 3:**
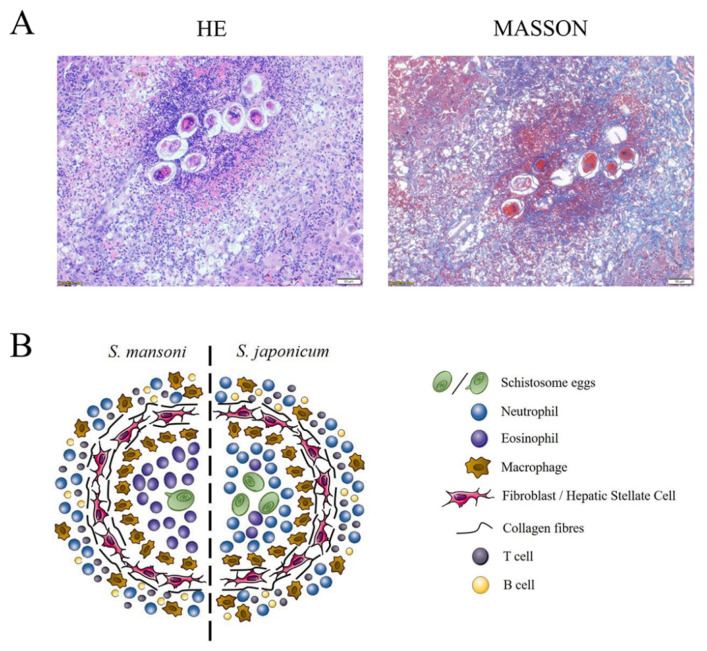
Representative histological images of hepatic granulomas of *S. japonicum* infection and illustration of granuloma composition of *S. mansoni* and *S. japonicum* infection. (**A**) Representative histology of *S. japonicum*-induced granuloma in BALB/c mice at 6 weeks post-infection. Formalin-fixed, paraffin-embedded liver sections stained with H&E and Masson. Scale bars, 50 mm. (**B**) Major cell populations of *S. mansoni* and *S. japonicum* infection. In *S. mansoni* infection, a granuloma is formed by a single egg and has an eosinophil dominant core. In S japonicum infection, a granuloma is formed by a cluster of eggs and the neutrophil as the dominate cell type. Abbreviation: HSC, hepatic stellate cell.

The composition of schistosome-induced granulomas varies among species, as neutrophils are the predominant cell type of *S. japonicum*-induced hepatic granulomas, while *S. mansoni* targets eosinophils, neutrophils, and macrophages, with eosinophils accounting for 50–70% of all granulomas [74] (Figure 3). The difference in the proportions of immune cells in *S. japonicum*- and *S. mansoni*-induced hepatic granulomas is due to a glycoprotein released by *S. mansoni* eggs, known as the IL-4-inducing principle of schistosome eggs/α-1, which is not expressed by *S. japonicum* eggs [75]. The function of this protein is activated upon binding to chemokines, such as C-X-C motif chemokine ligand 8, which is associated with the chemotaxis of neutrophil, giving rise to the inhibition of neutrophil infiltration to the site of infection [76]. 

Generally, after infection, granulomas begin to engage with eosinophils, macrophages, and neutrophils around the trapped egg, which further induces inflammatory responses [77]. In response to tissue injury, HSCs are then stimulated to generate ECM to induce fibrotic responses that contribute to the formation of an extra fibrotic zone [74]. Macrophages, eosinophils, and neutrophils accumulate at the periphery of the granuloma prior to infiltration [71]. At a later stage, several granule proteins involved with collagen degradation and reabsorption are released, which leads to remodeling of the impaired tissues and modulation of the pathology of granulomas [9,77]. During granuloma formation, neutrophils not only express proinflammatory cytokines, such as TNF- α, in the granuloma core, but also factors involved in collagen degradation and remodeling, such as matrix metalloproteinase-9 [77]. Besides, macrophages and HSCs also play pivotal roles in granuloma formation. HSCs are the main source of collagen in several hepatic fibrotic diseases other than schistosome-induced hepatic fibrosis, such as alcoholic liver disease, chronic viral hepatitis, and nonalcoholic fatty liver disease [71,78,79]. Storage of vitamin A is the primary function of quiescent HSCs, while activated HSCs in the fibrotic zone serve as an indicator of collagen deposition in hepatic schistosomiasis, which can be detected by immunostaining for the HSCs markers glial fibrillary acidic protein and α-smooth muscle actin (α-SMA) [80]. Furthermore, subsequent associations of activated HSCs with the NF-κB and transforming growth factor (TGF)/Smads pathways promote hepatic inflammation and fibrosis, which are discussed in greater detail later in this review [81,82]. Macrophages are vital to the pathogenesis of schistosomiasis and account for more than 30% of cells in liver granulomas induced by *S. mansoni* [83]. However, information on the proportion of macrophages in *S. japonicum*-induced granulomas is limited, thus warranting further exploration. Macrophages can be classified into two groups with distinct biological features: alternatively activated macrophages, which enhance fibrogenesis of fibroblasts by providing profibrogenic factors, and classically activated macrophages, which promote inflammation in the early stage of liver injury and inhibit fibrogenesis of fibroblasts by releasing antifibrogenic or fibrolytic factors in the late stage of hepatic fibrosis [84]. 

The dynamic development of liver granuloma is regulated by neutrophils as the primary cell type and serves a dual purpose, as well as HSCs and macrophages [9,85]. The collaboration of various cell types can lead to the formation of hepatic fibrosis and subsequent hepatosplenomegaly, portal hypertension, ascites, and esophageal varices, which can be fatal to the schistosomiasis host [2,6]. An illustration of the granuloma composition and representative histological images of murine hepatic granulomas due to *S. japonicum* infection are shown in Figure 3.

## 4. HMGB1 and Schistosome-Induced Liver Damage

Chronic hepatic inflammation is closely related to fibrosis in virtually all liver diseases and experimental models of fibrogenesis. However, the molecular relationship between inflammation and hepatic fibrosis remains unclear. Following the formation of hepatic granulomas induced by chronic schistosomiasis, HMGB1 is released from stressed or activated cells and exhibits pleiotropic functions in hepatic inflammation and fibrosis. In the following sections, the role of HMGB1 in schistosome-induced liver damage is discussed in more detail.

### 4.1. HMGB1 and Liver Inflammation

Animal studies have found that HMGB1 triggered systemic inflammatory diseases, including mastitis, sepsis, arthritis, epileptogenesis, necrotizing enterocolitis, acute lung injury, traumatic brain injury, and liver injury [86]. Elevation of HMGB1 levels in plasma and extracellular tissues is consider to promote inflammation. Structurally, the TLR-binding site of the B-box domain of HMGB1 initiates the HMGB1-induced inflammatory activities, whereas the A-box antagonizes this function [15]. Previous studies have demonstrated the role of the HMGB1/TLR4 signaling pathway in several HMGB1-mediated inflammatory diseases. For example, Lian et al. [87] reported that upon release, HMGB1 acted as a late-phase mediator of lipopolysaccharide-induced depression, which could be abrogated by a TLR4 inhibitor, while Yu et al. [88] found out that the application of glycyrrhizin (GL), a natural antagonist of HMGB1 extracted from licorice root, inhibited activation of the TLR4/NF-κB signaling pathway in necrotizing enterocolitis. In ischemia-reperfusion injury, inhibition of HMGB1 attenuates the expression of downstream proinflammatory cytokines activated by the TLR4/NF-κB signaling pathway [42].

In liver injury, activated HSCs can express TLR2, TLR3, TLR4, and TLR9. The interaction between TLR4 and liver inflammation has been widely reported [42,43,44]. For instance, TLR4 promotes early alcohol-induced liver injury by responding to increasing levels of circulating endotoxins [89]. The ability of TLR4 to inhibit the replication of hepatitis B virus (HBV) suggests that TLR4 is associated with chronic HBV infection [90]. Besides, TLR4 can induce hepatocellular carcinoma by increasing the number of lymphocytes and follicular helper-like cells residing in the liver and the production of pro-inflammatory cytokines [91]. In an animal model of carbon tetrachloride-induced hepatic fibrosis, TLR4 was reported to activate the Myd88/NF-κB pathway in HSCs [92]. The NF–κB complex consists of p50 and p65 dimers, which are retained in the cytoplasm by binding to the inhibitory protein IκB [93]. Once activated, NF-κB is translocated from the cytoplasm to the nucleus, where it binds to the κB site of target genes and induces the stimulation of inflammatory cytokines, which can result in liver damage [94]. Seki et al. [92] found that as compared with wild-type mice, the expression of the proinflammatory cytokines TNF-α and IL-6 was significantly reduced in Myd88-deficient mice. An interesting recent study reported that inhibition of HMGB1/TLR4 intracellular signaling decreased production of NF-κB, TNF-α, and IL-6 in cholestatic liver inflammation, which further confirms the close interactions among HMGB1, TLR4, and NF-κB in the promotion of liver inflammation [95].

HMGB1 and the NF-κB pathway are tightly linked through TLR4. Activation of HMGB1 and downstream signaling pathways contribute to inflammation and play pivotal roles in schistosome infection of various organs, including the liver. However, the direct connection between HMGB1 and schistosome-induced liver inflammation is poorly understood. In a study conducted by Vicentino et al. [19], high levels of HMGB1 were observed in the sera of mice and patients infected with *S. mansoni*. In order to comfirm the involvement of HMGB1 in schistosome-induced liver inflammation, mice were treated with the HMGB1 inhibitors GL and the synthetic compound 3-chloro-5-(4-pyridyl)-4,5-dihydroisoxazole (DIC) [19]. The results show that inhibition of HMGB1 leads to healthier livers and higher survival rates with down-regulation of the proinflammatory cytokines IL-6, IL4, IL-5, IL-13, and IL-17A and up-regulation of the anti-inflammatory cytokine IL-10 [19], demonstrating the important role of the HMGB1/TLR4 signaling pathway in schistosome-induced liver inflammation. In this regard, due to the contributing role of HMGB1 in various inflammatory diseases, toxic and antigenic substances released by schistosome eggs may directly or indirectly trigger the proinflammatory function of HMGB1, which leads to downstream activation of the TLR4/NF-κB signaling pathway.

### 4.2. HMGB1 and Liver Fibrosis

Liver fibrosis is a wound-repair process in response to liver injury and inflammation, which can be induced by a variety of persistent factors such as toxins, chronic viral infection, long-term alcohol abuse, and chronic parasitic disease [96,97,98]. Despite the complex etiology, liver fibrosis is characterized by the increased deposition of ECM involving activated HSCs [99]. Damaged HSCs exhibits a proliferative and fibrogenic myofibroblast-like phenotype, which can be confirmed by the expression of α-SMA [100]. Recent studies have suggested that activated HSCs accumulate in the periphery of murine and human granulomas induced by *S. japonicum* egg infections and are identified as effector cells that further contribute to granuloma-associated fibrosis [80]. Therefore, it is important to understand the physiological relationship between granuloma formation and down-modulation of HSCs.

HMGB1 is a contributing factor in hepatic fibrosis. Damage to the liver parenchyma triggers the release of HMGB1, resulting in the transdifferentiation of HSCs into scar-forming liver myofibroblasts [101]. Activated HSCs then secrete matrix proteins, such as α-SMA and collagen type I, into the extracellular space, leading to liver scar formation [102]. Structurally, HMGB1-driven α-SMA upregulation in cultured HSCs is reportedly dependent on RAGE [103]. Serum HMGB1 levels are significantly upregulated in patients with chronic HBV infection and in those with mild fibrosis as compared with severe fibrosis [104]. In a mouse model of carbon tetrachloride-induced liver fibrosis, the increase in serum HMGB1 was proportional to the elevation of TGF-β1 and collagen deposition during fibrogenesis [103]. More importantly, in the same study, HMGB1 stimulated the proliferation of HSCs in vitro, up-regulated the synthesis of α-SMA, and triggered Smad2 phosphorylation through the TGF-β1/Smad signaling pathway [103].

TGF-β1 is the most common TGF-β isoform in the mammalian liver [81,105]. Previous studies have demonstrated that the biological effects of TGF-β1 in cellular proliferation, differentiation, and fibrogenesis are dependent on phosphorylation of the downstream mediators Smad2 and Smad3, which existed in complexes and negatively regulated by the inhibitory mediator Smad7 [106,107]. In addition, as one of the first signals to activate quiescent HSCs during liver fibrosis, TGF-β1 is released by necrotic hepatocytes upon interactions with the cell surface receptors TβRI and TβRII, both of which are expressed by HSCs [108]. Hence, it can be concluded that the TGF-β1/Smad pathway is a key mediator of HSCs during hepatic fibrosis. Interestingly, HMGB1 and the TGF-β1/Smad pathway also seem to be relevant in renal and lung fibrosis [109,110]. However, further studies are needed to explore the interaction between HMGB1 and TGF-β1 in HSCs.

The pathogenesis of schistosome-induced liver fibrosis involves multiple pathways [71]. Studies have shown that HMGB1 was predominantly localized in the cytoplasm of hepatocytes surrounding schistosome-induced granulomas in the livers of mice with acute schistosomiasis as well as the nuclei of hepatocytes during chronic infections [19]. Interestingly, based on data of other fibrotic disease, this fibrotic signal might not be triggered by HMGB1 via TLRs. The receptor-specific selectivity of a fibrotic response appears to differ from liver inflammation, where HMGB1 interacts with both TLRs and RAGE. Arriazu et al. [111] suggested that HMGB1-elicited signaling in fibrotic disease involved not only the TGF-β1/Smad signaling pathway, but also the RAGE/phosphoinositide 3-kinase (PI3K)/AKT or RAGE-ERK pathway. As a fundamental regulator of signal transduction in protein synthesis, AKT can be transformed into a phosphorylation state via stimulation by activated PI3K [112], which then promotes the release of the inflammatory cytokines TNF-α, IL-1β, and IL-18, thereby stimulating the inflammatory cascade response resulting in damage to various organs and tissues [101].Therefore, HMGB1 seems to play a pivotal role in liver fibrosis by activating HSCs mainly through RAGE receptors to initiate collagen synthesis and deposition in liver fibrosis.

**Figure 4:**
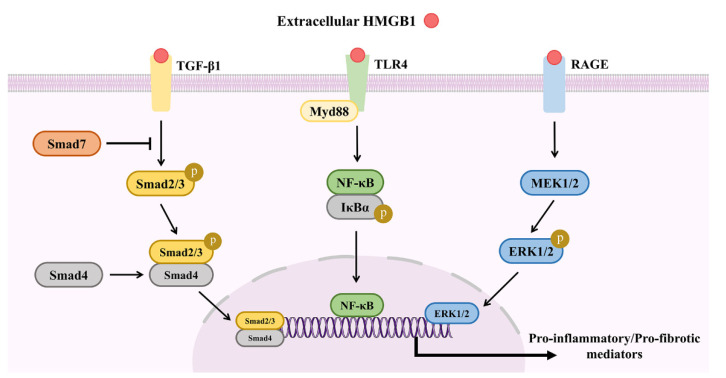
Signaling pathways involved in HMGB1 activities during schistosome-induced liver damage. During schistosome-induced liver damage, extracellular HMGB1 can trigger activation of the TGF-β1/Smad, TLR4/MyD88/NF-κB and RAGE/MEK/ERK in HSCs, promoting the production of pro-inflammatory or pro-fibrotic cytokines.

A recent report of HMGB1 in schistosome-induced liver fibrosis found that serum levels and relative mRNA expression of HMGB1 were significantly higher in the livers of the *S. japonicum*/*S. mansoni*-infection group, while the expression levels of the pro-inflammatory and fibrogenic cytokines IFN-γ, TGF-β1, and IL-6 were reduced [19,20]. Based on the close relationship between HMGB1 and TGF-β1 and the PI3K/AKT pathway in various fibrotic diseases, the HMGB1/TGF-β1 and HMGB1/RAGE/PI3K/AKT pathways may have a synergistic effect in granuloma-associated liver fibrosis, although little has been reported about any direct connections, such as whether upregulation of the TGF-β1/Smad pathway is mediated by the RAGE binding site of HMGB1. Since elevated serum and tissue levels of HMGB1 have been correlated with inflammation- and/or fibrosis-mediated pathologies in fibrosis of the liver, lung, and kidney, HMGB1 should be further investigated as a potential drug target. An illustration of the signaling pathways involved in the activities of HMGB1 during schistosome-induced liver damage is presented in Figure 4.

## 5. HMGB1 as a Therapeutic Target

Increasing evidence indicates that as a multi-functional mediator, HMGB1 contributes to a variety of systemic diseases, including ischemia-reperfusion injury, enterocolitis, sepsis, and liver injury, indicating the potential of HMGB1 as a therapeutic target [42,86,88,101]. Recently, under the worldwide threat of sudden acute respiratory syndrome (SARS-CoV-2), the etiological agent of COVID-19 disease, the progression from acute respiratory failure to sepsis has been correlated with the release of HMGB1 [113]. Wei et al. [114] proposed that HMGB1 regulates the expression of angiotensin-converting enzyme 2, which is essential for the entry of SARS-CoV-1 (highly pathogenic zoonotic CoVs), SARS-CoV-2 (highly pathogenic zoonotic CoVs), and human coronavirus NL63. Emerging studies have indicated that the inhibition of HMGB1 can improve the courses of all related diseases. For instance, knockout of HMGB1 protected against cell death induced by SARS-CoV-2 and the degree of protection were correlated with the level of HMGB1 [114]. Inhibition of HMGB1 was found to improve intestinal inflammation in necrotizing enterocolitis by inhibiting NLR pyrin domain containing 3 via the TLR4 and NF-κB signaling pathways [88]. Besides, HMGB1-neutralizing antibodies relieved liver inflammation and functional injury in the early stages of nonalcoholic fatty liver disease [115]. Another study demonstrated that HMGB1-neutralizing antibodies rescued cystic fibrosis-induced macrophage dysfunction and reduced lung inflammation induced by Pseudomonas aeruginosa [116].

This article highlighted the emerging roles of HMGB1 in liver diseases. Numerous studies have also suggested HMGB1 as a therapeutic target for liver diseases by blocking the release of HMGB1 or inhibiting the activity of extracellular HMGB1. GL is a glyco-conjugated triterpene produced by Glycyrrhiza inflata, which is an anti-inflammatory used for the treatment of chronic HBV infection [117]. Masahito et al. [118] found that GL reduced HMGB1 expression in Kupffer cells, suggesting that GL has therapeutic potential to prevent liver injury. A further study reported that the interactions between GL and the A box/B box of HMGB1 antagonized the inflammatory response [119]. Recently, GL was also considered a viable therapeutic option for SARS-CoV-2 infection due to the dual ability to concomitantly inhibit virus replication and diminish the production of proinflammatory mediators [120]. In schistosome-induced liver disease, Vicentino et al. [19] used GL to treat mice infected with *S. mansoni* from 21 to 56 dpi, which significantly reduced the area of the granuloma as well as serum cytokine levels. However, GL had no significant effect on the egg loads in the intestine, suggesting that GL had no schistosomicidal activity [19]. Similar results were obtained with the HMGB1 inhibitor DIC [94]. Interestingly, sodium butyrate, a deacetylase inhibitor, which conveys a protective effect in certain inflammatory diseases, such as sepsis and lipopolysaccharide-induced acute lung injury, can also reduce hepatic granulomas and fibrosis induced by *S. japonicum* by inhibiting HMGB1 expression [20]. Ge et al. [21] used small interfering RNA to directly inhibit HMGB1 expression in rat HSCs, which inhibited activation of HSCs.

Collectively, these data indicate a pro-fibrotic role of HMGB1 in schistosome-induced liver granulomas. Inhibiting the expression of HMGB1 by interference methods or antagonists, or suppressing downstream signaling pathways, such as HMGB1/RAGE, are potential strategies to treat schistosome-induced liver fibrosis.

## 6. Concluding Remarks

Humans and animals with chronic schistosome infection can develop chronic liver disease, which develops over many years and often decades. Without prompt treatment, once organ function has been significantly compromised and is irreversible, patients usually present with advanced disease. Studies of early biomarkers have focused on the status of liver disease. HMGB1 is a wildly expressed and conserved protein with multiple functions, including regulation of liver inflammation and fibrosis. Treatment to inhibit the secretion and function of HMGB1 could provide significant protection against liver inflammation and fibrosis. To date, limited studies have investigated the relationship between HMGB1 and schistosome-induced liver disease. Given that the lives of hundreds of millions of people are threatened by schistosomiasis, further research efforts should focus on HMGB1 as a potential therapeutic target for schistosome-induced liver disease. Moreover, the potential collaboration of PZQ with HMGB1 inhibitors as a treatment for schistosomiasis should be explored. In addition, future studies are warranted to determine whether HMGB1 is involved in the pathogenesis of *S. haematobium*-induced disease and the function HMGB1 in schistosomes. Regardless, the discoveries discussed in this review describe the current knowledge of the roles of HMGB1 in liver disease, with an emphasis on schistosomiasis. Reports in this area pose profound questions regarding the exploration and treatment of liver inflammation and fibrosis, both parasitic and nonparasitic in origin.
